# Postural Hypotension Associated with Nonelastic Pantyhose during Lymphedema Treatment

**DOI:** 10.1155/2014/536126

**Published:** 2014-07-06

**Authors:** Jose Maria Pereira de Godoy, Daniel Zucchi Libanore, Maria de Fatima Guerreiro Godoy

**Affiliations:** ^1^Cardiology and Cardiovascular Surgery Department, Medicine School in São José do Rio Preto (FAMERP), Avenida Constituição 1306, 15025120 São José do Rio Preto, SP, Brazil; ^2^Research Group in Godoy Clinic, Avenida Constituição 1306, 15025120 São José do Rio Preto, SP, Brazil; ^3^Medicine School in São José do Rio Preto (FAMERP) and Research Group in Godoy Clinic, Avenida Constituição 1306, 15025120 São José do Rio Preto, SP, Brazil

## Abstract

The case of a 72-year-old female patient with elephantiasis is reported. The patient was submitted to two surgeries to remove the edema. After surgery, the leg again evolved to elephantiasis and eventually she was referred to the Clinica Godoy for clinical treatment. Intensive treatment was carried out (6 to 8 hours per day) and the patient lost more than 70% of the limb volume within one week. After this loss, the volume was maintained using grosgrain compression pantyhose for 24 hours per day. During the return appointment, the patient suffered from systemic hypotension (a drop of more than 30 mmHg within three minutes) while she was standing after removing the stocking. A further investigation showed that the symptoms only appeared when the stocking was worn for 24 hours. Thus, the patient was advised to use the stocking only during the day thereby avoiding the symptoms of hypotension.

## 1. Introduction

Rapid cardiovascular adjustment is essential to avoid orthostatic hypotension in the passage from the decubitus to the standing position; a response is required within seconds [1, 2]. Orthostatic hypotension is defined as a drop of at least 20 mmHg of systolic pressure or 10 mmHg of diastolic pressure within three minutes when changing from the supine or sitting position to the standing position [3]. Dizziness, blurred vision, weakness, nausea, palpitations, headache, syncope, and chest pains are the most commonly reported symptoms. Ineffective adrenergic vasoconstriction provides an inadequate response to adjust the systemic arterial pressure [4].

Studies suggest that orthostatic stress evokes regional differences in cerebral blood flow with possible differences in the carotid dynamics between the two vascular brain regions leading to acute changes in blood pressure [5, 6]. Graduated compression stockings might affect the sympathoadrenergic variability and heart rate variability in response to rest and after strenuous exercise by individuals in wheelchairs with spinal cord injury [7].

Nonelastic compression mechanisms are recommended in the treatment of lymphedema. The daily clinical experience shows that the maintenance of compression at night in the initial stage of the treatment of severe lymphedema (grades II and III) allows maintenance of the volume reductions achieved during the day. Thus, the use of compression at night is frequently indicated. However, one patient started to suffer from postural hypotension. The aim of this study is to report on a case of postural hypotension after the continuous use (24 hours/day) of nonelastic pantyhose for the treatment of lymphedema.

## 2. Case Report

We report on the case of a 72-year-old female patient who since the age of 45 has had lymphedema that evolved to elephantiasis. She was submitted to two surgeries to remove tissue related to the elephantiasis, but the lymphedema again progressed to elephantiasis during the years that followed. Eventually, the patient was referred to the Clinica Godoy for intensive treatment including mechanical lymphatic therapy, cervical stimulation, nonelastic compression stocking (grosgrain), and manual lymphatic therapy (Pereira de Godoy and de Fatima Guerreiro Godoy [8], and Pereira de Godoy et al. [9]). This technique uses a hand-made low-stretch compression stocking of a cotton-polyester fabric (Grosgrain) [10]. The patient, with a height of 81 kg, had a body mass index (BMI) of 34.6 kg/m^2^ before starting treatment which dropped to 32.6 kg/m^2^ after treatment. There was a 70% reduction in the volume of the leg within five days of treatment after which the use of a grosgrain nonelastic stocking was prescribed for 24 hours per day. On the patient's return visit to the clinic after 15 days of using the pantyhose, she presented with postural hypotension after removing the stocking for a physical evaluation. The reduction in systemic blood pressure, assessed in the standing position with measurements at one-minute intervals, showed a reduction of more than 30 mmHg within three minutes. With the drop in blood pressure, the patient had symptoms of hypotension and was placed in the supine position with improvement of the symptoms and the pressure returning to normal. Blood pressure measurements were repeated in the standing position and the patient again had hypotensive symptoms. The patient was advised not to use pantyhose at night and when it was taken off at the next appointment the patient had no symptoms of hypotension. Again the use of the pantyhose was reintroduced for 24 hours per day and again on removing it the patient had symptoms of hypotension with a 30 mmHg drop in blood pressure in 3 minutes. Finally, the patient was advised not to wear the pantyhose at night and the postural hypotension was definitively cured. This study was approved by the Research Ethics Committee of the Medicine School in São José do Rio Preto (FAMERP) number 144.958/12.

## 3. Discussion

The current study reports on postural hypotension with the continuous use (24 hours per day) of nonelastic grosgrain pantyhose. This situation, associated with compression therapy, has not been described in the literature previously. However, one method described to treat postural hypotension uses elastic stockings [6] although there is no recommendation to use the stockings for 24 hours per day as in this case.

In this study, the age of the patient (72 years old) may have contributed to her condition. Other known causes of hypotension, including dehydration, blood loss, neurological disorders, other cardiovascular and endocrine causes, and certain classes of medicines, were not present in this patient. The improvement in the symptoms with the removal of the compression garment at night and worsening of the hypotension when it was reintroduced strongly suggests that the pantyhose contributed to these symptoms. About three years ago, a 29-year-old patient had very similar symptoms, but at that time the drop in pressure was not thought to have been an effect of the treatment. Hence, this is the second case and serves as a warning about this danger.

Grosgrain stockings are nonelastic compression mechanisms with a resting pressure of between 10 and 30 mmHg and thus they provide a good continuous compression. This complication is not seen with knee-length grosgrain stockings or even with pantyhose when used only during the daytime. Thus, this study is a warning about the possibility of hypotension when pantyhose is used 24 hours per day.

Orthostatic capacity is an important index for evaluating cardiovascular regulation. Reduced orthostatic tolerance may be associated with cardiac dysrhythmias, myocardial injury with ischemia, diminished cardiac and vascular function that appear to include reductions in circulating blood volume, compromised hemodynamic responses to central hypovolemia, and decreased cerebral and muscle blood flow [11–13]. We speculate that changes in any of these parameters could have contributed to orthostatic changes upon standing of this patient. However, we did not measure any of these variables. Further research should be directed at examining the exact mechanism that contributes to the orthostatic intolerance in subjects with grosgrain compression pantyhose.

The hypothesis for the occurrence of hypotension in this case is interference in the sympathetic nervous mechanisms involving the vascular system. The continuous compression (24 hours per day) may inhibit sympathetic reflexes thereby interfering in the control of pressure.
